# miR‐204 downregulates EphB2 in aging mouse hippocampal neurons

**DOI:** 10.1111/acel.12444

**Published:** 2016-01-22

**Authors:** Chand Parvez Danka Mohammed, Hwanseok Rhee, Bong‐Kwan Phee, Kunhyung Kim, Hee‐Jin Kim, Hyehyeon Lee, Jung Hoon Park, Jung Hee Jung, Jeong Yeon Kim, Hyoung‐Chin Kim, Sang Ki Park, Hong Gil Nam, Keetae Kim

**Affiliations:** ^1^ Center for Plant Aging ResearchInstitute for Basic Science (IBS)Daegu711‐873Korea; ^2^ Department of New BiologyDGISTDaegu711‐873Korea; ^3^ Department of Life SciencesPOSTECHPohang790‐784Korea; ^4^ Macrogen Inc.Seoul153‐781Korea; ^5^ Korea Research Institute of Bioscience and BiotechnologyOchang363‐883Korea

**Keywords:** aging, EphB2, hippocampus, miRNA204, NMDA receptor

## Abstract

Hippocampal synaptic function and plasticity deteriorate with age, often resulting in learning and memory deficits. As MicroRNAs (miRNAs) are important regulators of neuronal protein expression, we examined whether miRNAs may contribute to this age‐associated decline in hippocampal function. We first compared the small RNA transcriptome of hippocampal tissues from young and old mice. Among 269 hippocampal miRNAs, 80 were differentially expressed (≥ twofold) among the age groups. We focused on 36 miRNAs upregulated in the old mice compared with those in the young mice. The potential targets of these 36 miRNAs included 11 critical Eph/Ephrin synaptic signaling components. The expression levels of several genes in the Eph/Ephrin pathway, including EphB2, were significantly downregulated in the aged hippocampus. EphB2 is a known regulator of synaptic plasticity in hippocampal neurons, in part by regulating the surface expression of the NMDA receptor NR1 subunit. We found that EphB2 is a direct target of miR‐204 among miRNAs that were upregulated with age. The transfection of primary hippocampal neurons with a miR‐204 mimic suppressed both EphB2 mRNA and protein expression and reduced the surface expression of NR1. Transfection of miR‐204 also decreased the total expression of NR1. miR‐204 induces senescence‐like phenotype in fully matured neurons as evidenced by an increase in p16‐positive cells. We suggest that aging is accompanied by the upregulation of miR‐204 in the hippocampus, which downregulates EphB2 and results in reduced surface and total NR1 expression. This mechanism may contribute to age‐associated decline in hippocampal synaptic plasticity and the related cognitive functions.

## Introduction

Aging is often associated with cognitive decline and increased propensity for neurological diseases, leading to a lower quality of life in the elderly (Hedden & Gabrieli, 2004). The hippocampus is necessary for explicit learning in humans, and hippocampal synapses exhibit robust forms of long‐lasting associative synaptic plasticity that appear necessary for certain forms of learning in animals (Burgess *et al*., 2002). There have been numerous studies on the cellular and molecular mechanisms of cognitive impairment resulting from hippocampal aging and dysfunction (Driscoll *et al*., 2003; Chételat *et al*., 2013). Hippocampal aging is associated with a little change in the hippocampal gross structure or volume; it is associated with biochemical changes in the expression of hundreds of genes involved in neuronal signaling and synaptic plasticity (Blalock *et al*., 2003). Alzheimer disease (AD), one of the most devastating age‐related neurological diseases, involves functional impairment of the hippocampus, and older age is the greatest risk factor for idiopathic AD. It was recently shown that the transmembrane receptor tyrosine kinase EphB2 is depleted in the hippocampus of an AD mouse model and that reversing this depletion can rescue cognitive function (Cissé *et al*., 2011). Binding of EphB2 to its physiological ligand ephrin initiates signaling pathways critical for neuroplastic processes such as axon guidance, angiogenesis, and long‐term potentiation, a form of associative synaptic plasticity observed at hippocampal synapses (Klein, 2008).

MicroRNAs (miRNAs) repress protein expression by mainly binding to the 3′ untranslated region (UTR) of their target mRNAs and by destabilizing the mRNA or arresting translation (Huntzinger & Izaurralde, 2001; Ambros, 2004; Lewis *et al*., 2005). Approximately 50% of mammalian miRNAs are expressed in the brain (Krichevsky *et al*., 2003; Somel *et al*., 2011), many of which have critical roles in neurogenesis and neuronal development (Giraldez *et al*., 2005; De Pietri Tonelli *et al*., 2008). A number of hippocampal miRNAs regulate neuronal activity by targeting their downstream genes (Eacker *et al*., 2011; Juhila *et al*., 2011). For instance, in the miRNA‐mediated synaptic plasticity regulating pathway, miR501 (Hu *et al*., 2015), miR223 (Harraz *et al*., 2012) and miR134 (Jimenez‐Mateos *et al*., 2012) target GluR1, GluR2 and NR2B, and DHX36, respectively. Furthermore, small RNA transcriptome analysis in the whole brain of mouse revealed that miRNAs are potentially involved in the regulation of brain aging (Eacker *et al*., 2011). Hippocampal aging in rats and mice is associated with changes in the expression of many miRNAs (Inukai *et al*., 2012), suggesting that miRNAs contribute to cognitive decline by suppressing hippocampal proteins necessary for the neurocellular and synaptoplastic processes underlying learning and memory.

To test this notion, we profiled changes in the small RNA transcriptome of mouse hippocampus during aging (2–18 months). Age‐upregulated miRNAs were identified and one, miR‐204, was chosen as a promising candidate for inducing age‐related hippocampal dysfunction because it has been linked to EphB2 suppression; EphB2 was in fact downregulated in aged mouse hippocampus. Moreover, EphB2 is a known regulator of NMDA receptor subunit NR1 surface expression. We thus tested the effects of miR‐204 overexpression on EphB2 and NR1 surface expression in mouse hippocampal neurons. We also examined whether miR‐204 induces senescence in fully matured hippocampal neurons and observed an increase in the percentage of p16‐positive cells. This report showing miR‐204‐induced downregulation of EphB2 and NR1 in hippocampal neurons provides an important clue to the molecular mechanisms of age‐associated hippocampal dysfunction and associated cognitive decline.

## Results

### Hippocampal miRNAs are differentially regulated during aging

**Figure 1:**
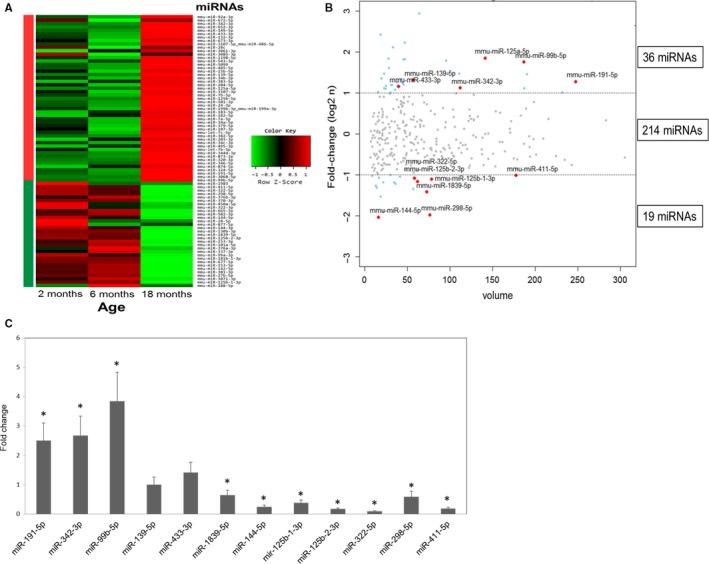
MicroRNAs differentially expressed in aged hippocampus of mouse. (A) Heat map of 80 miRNAs showing ≥ twofold change in the hippocampus between any two of the three age groups. Small RNA samples were pooled out of eight hippocampi from four individual mice for each age group. This sample was used for sequencing as a single library and subsequent bioinformatical analysis. (B) Scattered volume plot of miRNAs differentially expressed between the 2‐ and 18‐month‐old hippocampi with ≥ twofold change. The size of the marker represents the magnitude of expression according to the formula $\sqrt{\left(\mathrm{RPM}2m)*(\mathrm{RPM}18m\right)}$. The miRNAs marked in red were selected for qPCR validation. (C) qPCR validation of representative miRNAs differentially expressed between the 2‐ and 18‐month‐old hippocampi. miRNA levels were measured from three independent samples. Shown are the average and standard deviation for each miRNA, *P < 0.05. This result reflects the reliability of NGS data.

To assess whether changes in miRNA expression contribute to age‐related hippocampal dysfunction, we profiled the small RNA transcriptome of C57BL/6J male mice at the ages of 2, 6 and 18 months. The total number of filtered read counts was near or greater than 5 million for each stage. Known miRNA reads were between 1.5 and 2.0 million, and non‐miRNA reads were between 2.7 and 3.3 million for each stage (Table S1). miRNAs with less than 10 read counts were excluded from further analyses. The expression profiles of miRNAs in the hippocampus at all three ages are shown in Table S2. We identified 269 miRNAs expressed in the hippocampus, accounting for approximately 30% of all miRNAs identified in mice, in accordance with previous reports showing that the brain (and particularly the hippocampus) is enriched in miRNAs (Noren Hooten *et al*., 2010; van Spronsen *et al*., 2013). We then identified miRNAs differentially expressed among the three age groups. Heat map analysis revealed that 80 of the 269 hippocampal miRNAs were differentially (twofold or greater) expressed when we combined the miRNAs differentially expressed between any two ages (Fig. 1A). Of these, 49 were upregulated and 31 were downregulated. The heat map (Fig. 1A) and cluster analyses (Fig. S1) of the differentially expressed miRNAs showed that most expression changes were observed in the 18‐month‐old mice, whereas the expression changes between the mice aged 2 and 6 months were minimal. This is in contrast to a previous report in the whole brain of mice, where more downregulated miRNAs were found during aging (Inukai *et al*., 2012). The greater proportion of differentially expressed miRNAs in the aged mice suggests that the downregulation of specific proteins contributes to age‐related deficits in the hippocampus. For further analysis of age‐associated miRNAs, we compared the expression of miRNAs in the 2‐ and 18‐month‐old mice (Fig. 1B). The differential expression of the miRNAs identified by small RNA‐seq data was confirmed by quantitative PCR analysis (Figs 1C and S2). Among the hippocampal miRNAs differentially expressed between 2 and 18 months, 36 were upregulated and 19 were downregulated over twofold in the aged mice (Fig. 1B). Furthermore, the expression levels of the upregulated miRNAs were uniformly higher than those of the downregulated miRNAs, suggesting a greater regulatory role of miRNA‐mediated translation suppression with age.

### Downregulation of Eph/ephrin pathway in aged hippocampus

To identify specific functions of these upregulated miRNAs in hippocampal aging, we searched the miRTarBase (http://mirtarbase.mbc.nctu.edu.tw) to find experimentally validated mouse targets. The targets genes were then subjected to pathway annotations using the Kyoto Encyclopedia of Genes and Genomes (KEGG). The most frequent targets of these miRNAs were cancer‐pathway genes (11 genes) (Table 1). The others included TGF‐beta and MAP kinase signaling pathway genes. As the number of experimentally validated targets is limited, we also searched for potential targets of these age‐upregulated miRNAs using TargetScan (http://www.targetscan.org/). The potential targets of these miRNAs included 136 cancer, 69 axon guidance and 67 insulin signaling pathway genes (Table S3). The high incidence of the insulin signaling pathway genes is consistent with that of a previous report (Inukai *et al*., 2012).

**Table 1:** Pathway annotations associated with experimentally validated targets of miRNAs upregulated in aged hippocampus

Gene ontology	Gene Count	*P*‐value
Pathways in cancer	11	4.30E‐07
TGF‐beta signalling pathway	7	1.40E‐06
MAPK signalling pathway	7	7.70E‐04
Wnt signalling pathway	5	3.60E‐03
Chronic myeloid leukema	4	4.20E‐03
Colorectal cancer	4	5.90E‐03
Cell cycle	4	1.70E‐02
Neurotrophin signalling pathway	4	1.80E‐02
Endometrial cancer	3	2.10E‐02
Acute myeloid leukaemia	3	2.50E‐02
Pancreatic cancer	3	3.90E‐02
Adherens junction	3	4.30E‐02
ErbB signalling pathway	3	5.40E‐02
Prostate cancer	3	5.80E‐02
GnRH signalling pathway	3	6.60E‐02
Toll‐like receptor signalling pathway	3	6.80E‐02
T‐cell receptor signalling pathway	3	9.30E‐02

**Figure 2:**
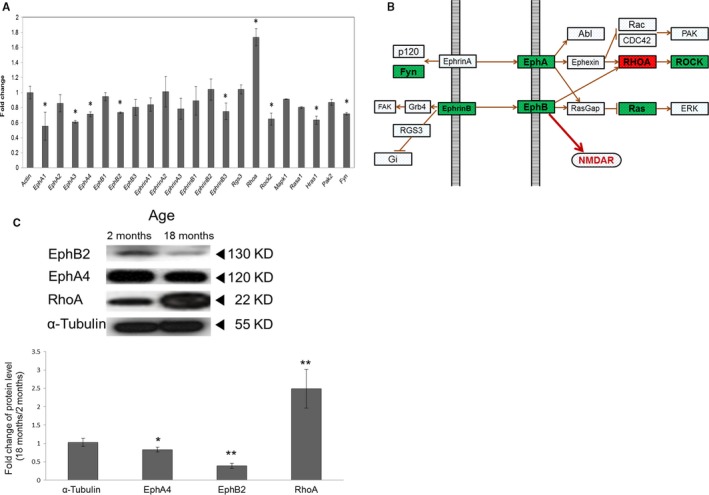
Downregulation of Eph/ephrin‐mediated axon guidance pathway genes in aged hippocampus. (A) qPCR validation of representative mRNAs in the Eph/ephrin‐mediated axon guidance pathway. mRNA levels were measured from three independent samples. Shown are the average and standard deviation for each mRNA, *P < 0.05. (B) Diagram depicting signaling relationships among the Eph/ephrin pathway genes, including those showing differential expression in hippocampus between the 2‐ and 18‐month‐old mice (red, upregulated in aged hippocampus; green, downregulated in aged hippocampus). (C) The protein levels of EphB2, EphA4, RhoA, and tubulin (internal control) in the 2‐ and 18‐month‐old hippocampi as measured by immunoblotting with antibodies specific to each protein. EphB2 protein level was downregulated by 60% in the aged hippocampus compared with that in the young hippocampus. *n* = 3 (three different animals), *P < 0.05 **P < 0.005.

The potential targets of the age‐upregulated miRNAs linked to axon guidance included 11 components of the Eph/ephrin signaling pathway (Table S4). We focused our analysis on the relationships between the age‐upregulated miRNAs and the expression of genes in the Eph/ephrin pathway as Eph signaling regulates synaptic plasticity and loss of EphB2 has been linked to age‐related cognitive dysfunction (Klein, 2008; Cissé *et al*., 2011). As many of the single components in the Eph/ephrin signaling pathway have multiple isoforms, we examined the expression of each gene by qRT–PCR. Of the 21 potential target genes in the Eph/ephrin signaling pathway (including isoforms), eight were downregulated (*P* < 0.05), 12 showed no significant change (*P* > 0.05), and only one was upregulated (*P* < 0.01) in the aged hippocampus (Fig. 2A). We further confirmed hippocampal expression of the ephrin ligand and receptor genes by qRT–PCR (Fig. S3A) and found that 7 of 14 putative miRNA target genes examined in the Eph/ephrin pathway were downregulated in the aged hippocampus. Of the 11 Eph/ephrin signaling components identified as possible targets (Table S4), six were downregulated, while RhoA, a downstream effector of Eph/ephrin signaling, was upregulated by 70% (Fig. 2A,B). We focused our interest on Eph receptor molecules in particular as they play the most critical roles in the maintenance of synaptic plasticity. The downregulated genes included critical Eph receptor molecules such as EphA4 and EphB2, showing approximately 30% and 25% decreases, respectively, in mRNA expression levels (Fig. S3A). EphA4 is involved in the regulation of synaptic plasticity (Murai *et al*., 2003; Filosa *et al*., 2009), and EphB2 controls both spine formation (Penzes *et al*., 2003) and synaptic plasticity (Grunwald *et al*., 2001, 2004; Henderson *et al*., 2001; Margolis *et al*., 2010). Thus, our results suggest that the age‐upregulated miRNAs downregulate the Eph/ephrin signaling pathway involved in synaptic function and plasticity.

### EphB2 is downregulated by miR‐204 in hippocampal neurons

Of the identified age‐upregulated miRNAs, miR‐204 is a promising regulator of Eph/ephrin signaling. First, miR‐204 targets EphB2 in glioma cells (Ying *et al*., 2014) and regulates retinal axon guidance through Efnb3 (ephrin ligand) and EphB2 (receptor) during eye development in medaka (Conte *et al*., 2014). We speculated that the age‐upregulated miR‐204 targets EphB2 mRNA and reduces EphB2 expression in aged hippocampus. Thus, we compared EphB2 mRNA and protein levels between the 2‐ and 18‐month‐old hippocampi and found that EphB2 protein was indeed 60% lower in the aged hippocampus (*P* < 0.005, *n* = 3), while EphB2 mRNA was reduced by 25% (*P* < 0.05) (Figs 2C and S3B).

**Figure 3:**
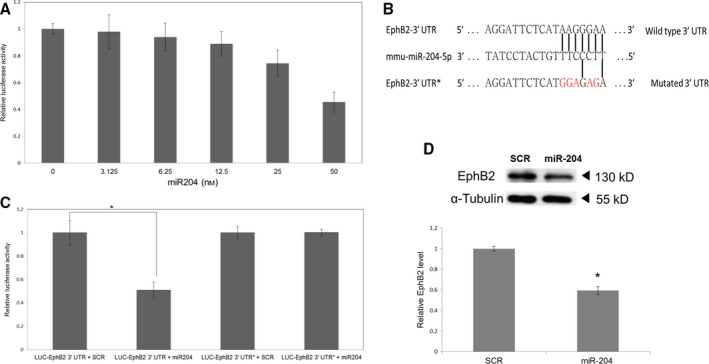
miR‐204 targets EphB2 in cultured primary hippocampal neurons. (A) miR‐204 represses the expression of luciferase‐Ephb2 3′ UTR in a dose‐dependent manner in primary hippocampal neurons. *N* = 3 independent cultures per treatment. Error bars indicate standard deviations. (B) The mutated 3′ UTR sequence of the luciferase‐Ephb2 3′ UTR* construct. Only the miRNA target sequences are shown. The mutated 3′ UTR contains five mismatches with miR‐204 (red). (C) Comparison of miR‐204‐induced luciferase repression in neurons transfected with wild‐type or mutant 3′ UTR sequences. SCR, Scrambled miRNA control. *n* = 3, error bars indicate standard deviations, *P < 0.005. (D) Effect of miR‐204 on EphB2 protein expression. Primary hippocampal neurons were transfected with miR‐204 mimic or scramble control miRNA (SCR). α‐tubulin was used as the gel loading control. Insert above: sample Western blot, *n* = 3. There was a significant decrease in EphB2 protein in neurons transfected with the miR‐204 mimic but not in those transfected with the scrambled control. Error bars indicate standard deviations, *P < 0.05.

We then tested whether miR‐204 can repress EphB2 expression in the HEK 293 cell line. For this assay, we generated a reporter construct (pLuc‐EphB2 3′ UTR) where the 3′ UTR of the EphB2 gene (miRNA‐bound region) was fused to the luciferase gene (LUC). Transfection with miR‐204 reduced luciferase activity by 35% (*P* < 0.05) compared with that by a scrambled control (Fig. S4). We then tested whether miR‐204 also represses EphB2 in hippocampal neurons. In primary hippocampal neurons 7 days *in vitro* (DIV) pretransfected with pLuc‐EphB2 3′ UTR, transfection of an miR‐204‐5p mimic resulted in a dose‐dependent decrease in luciferase activity (Fig. 3A), reaching 50% at 50 nm. We also tested the effect of the miR‐204 mimic in neurons expressing a version of pLuc‐Ephb2 3′ UTR with five point mutations in the miR‐204 binding sequence (Fig. 3B). In this case, the miR‐204 mimic did not induce a significant change in luciferase activity (Fig. 3C) indicating a specific interaction between the miR‐204 and Ephb2 3′ UTRs. In addition, the miR‐204 mimic repressed EphB2 protein expression by 40% (*P* < 0.05) in primary hippocampal neurons transfected at 2 DIV and harvested at 7 DIV (Fig. 3D).

### miR‐204 regulates surface expression of NR1 subunit of NMDA receptor

**Figure 4:**
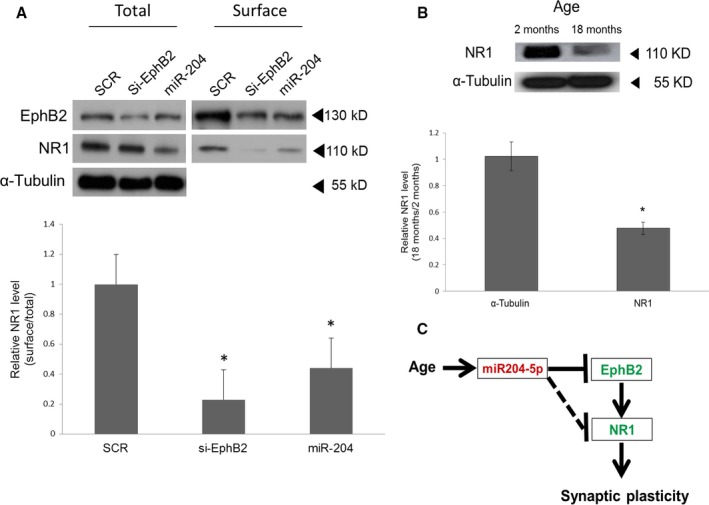
miR‐204 controls total and surface expression of the NMDA receptor NR1 subunit. (A) NR1 protein levels in 7 DIV hippocampal neurons after transfection with either scramble (SCR), siRNA against EphB2 or miR‐204 mimic (miR‐204) at 2 DIV. Biotinylated (surface) and total NR1 proteins were visualized by immunoblotting (top). α‐tubulin was used as a control. The absence of α‐tubulin in the surface fraction indicates clear separation of the intracellular and surface protein fractions. The surface fraction of NR1 was quantified from three independent experiments. Shown are the mean ratios of surface NR1 to total NR1. *P < 0.05, *n* = 3, error bars indicate standard deviations. (B) Reduced level of total NR1 protein in the aged hippocampus compared with that of the hippocampus as measured by Western blot (top). Shown at the bottom are the mean total NR1 levels quantified by densitometry from three independent experiments. *P < 0.05, *n* = 3, error bars indicate standard deviations. (C) Model of age‐associated loss of synaptic plasticity via miR‐204 upregulation. Red and green denote up‐ and downregulation of these genes, respectively, in the aged hippocampus. The solid line indicates miR‐204‐dependent NR1 regulatory pathways mediated by reduced EphB2 expression. The other pathway independent of EphB2 is indicated by the dotted line.

EphB2 is known to control the surface expression of the NMDAR NR1 subunit in hippocampal neurons (Cissé *et al*., 2011; Nolt *et al*., 2011), expression necessary for the induction of a robust form of long‐term hippocampal potentiation (Klein, 2008). In addition, EphB2 controlled the surface expression of NR1 in an AD mouse model, which could account for the reduced synaptic plasticity and learning deficits in these mice (Cissé *et al*., 2011). Collectively, these results suggest that miR‐204 downregulates the surface expression of the NR1 subunit in hippocampal neurons through EphB2. The effect of miR‐204 on the surface expression of the NR1 subunit was examined by surface biotinylation experiments in which primary hippocampal neurons were transfected with either the scrambled control or miR‐204 mimic at 2 DIV and harvested at 7 DIV for surface NR1 quantification. The total amount of NR1 protein was reduced by 38% upon transfection with the miR‐204 mimic, and the ratio of surface to total NR1 was decreased by 57% (Fig. 4A). siRNA against EphB2 as a positive control resulted in decrease in NR1 surface to total ratio by 77% compared with scramble (Fig. 4A), suggesting that miR204 is involved in the regulation of surface expression level of NR1 through EphB2.

The decrease in NR1 surface expression induced by miR‐204 transfection was likely due, at least in part, to the repression of EphB2 as miR‐204 represses EphB2, and EphB2 controls the surface expression of NR1 (Cissé *et al*., 2011; Nolt *et al*., 2011). However, EphB2 is not known to regulate the total cellular expression of NR1. Moreover, the miR‐204 reduction cannot be direct because NR1 mRNA does not have the 3′ UTR target sequence. Thus, there may also be a second EphB2‐independent pathway for the miR‐204‐mediated repression of NR1 that involves another intermediary target. Accordingly, the increased miR‐204 expression in the aged hippocampus could contribute to the approximately 50% reduction in NR1 compared with the 2‐month‐old hippocampus (Fig. 4B; Nolt *et al*., 2011; Liu *et al*., 2008). These different pathways are schematically summarized in Fig. 4C.

### miR‐204 induces senescence‐like phenotype in hippocampal neurons

**Figure 5:**
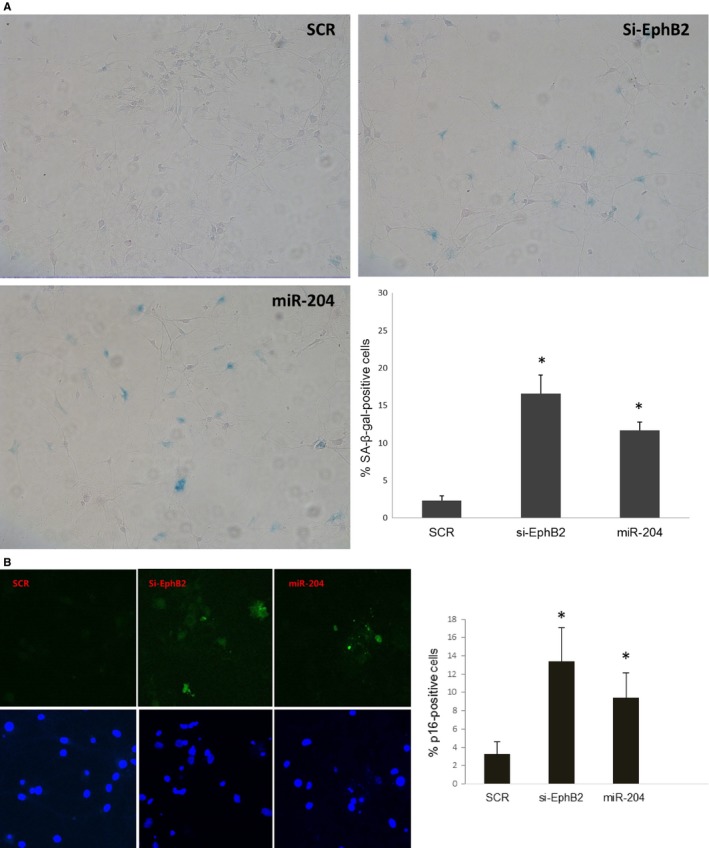
miR‐204 induces senescence‐like phenotype by repressing EphB2 in DIV 24 hippocampal neurons. (A) The appearance of increased SA‐ß‐Gal activity were analyzed and quantitated 21 days after transfection. Percentage of cells positive for the indicated marker is shown in histograms, which correspond to the mean. Error bars indicate standard deviations of three independent experiments from a total of at least 300 cells. *P < 0.05, *n* = 3 (B) Increased expression of senescent marker p16 in miR‐204‐transfected neurons. Error bars indicate standard deviations of three independent experiments from a total of at least 450 cells. *P < 0.05, *n* = 3.

As miR‐204 and EphB2 are increased and decreased upon aging, respectively, and aging is strongly associated with cellular senescence (Jurk *et al*., 2012), we asked whether miR‐204 and EphB2 are involved in cellular senescence of hippocampal neurons during aging. Long‐term (DIV 21 to DIV 40) primary neuronal cultures are a useful tool for the investigation of neuronal senescence (Bertrand *et al*., 2011). We thus performed senescence‐associated beta‐galactosidase (SA‐β‐gal) assay on primary cultured hippocampal neurons at DIV 24. miR‐204 mimic and siRNA against EphB2 resulted in 13% and 16% of SA‐β‐gal‐positive cells, respectively, whereas scramble showed about 2% of senescent positive cells (Fig. 5A). We further examined whether this increase in the percentage of SA‐β‐gal‐positive cells is associated with the induction of another senescent marker p16. We found that miR‐204 and siRNA against EphB2 resulted in 9% and 13% of p16‐positive cells, respectively, in contrast to scramble showing about 3% of p16‐positive cells (Fig. 5B). These data suggest that miR‐204 induces senescence by repressing EphB2 in aged primary cultured hippocampal neurons.

## Discussion

This report provides evidence that the upregulation of miR‐204 in aged hippocampus contributes to the age‐related downregulation of EphB2 and NR1, a possible mechanism for age‐related decline of hippocampal synaptic plasticity and associated cognition dysfunction. We demonstrate that (i) EphB2 is decreased in aged hippocampus concomitant with miR‐204 upregulation, (ii) EphB2 is a target of miR‐204 in hippocampal neurons and (iii) miR‐204 reduces both total NR1 expression and surface expression in hippocampal neurons. As EphB2 signaling is linked to surface expression (Cissé *et al*., 2011; Nolt *et al*., 2011) but not total NR1 expression, there may be two miR‐204‐dependent NR1 regulatory pathways, one mediated by reduced EphB2 expression and the other independent of EphB2 as indicated by the dotted line (Fig. 4C).

There is voluminous evidence that deficient NMDAR‐dependent LTP results in learning and memory impairments (David Sweatt, 2009) and that hippocampal LTP declines with age (Lynch, 2010; Kumar, 2011). Moreover, the rapid recruitment of NMDA receptor subunits can reduce the threshold for LTP, a process that appears to be dependent on dendritic mRNA translation (Swanger *et al*., 2013). Thus, the loss of critical signals involved in maintaining NMDAR surface numbers and LTP threshold through age‐dependent upregulation of miRNAs may be responsible, at least in part, for the age‐related decline in cognitive function. However, miR‐204 targets many other biological pathways including stemness and cell migration. In total, 269 miRNAs were expressed with read counts over 10 in the 2‐, 6‐, and 18‐month‐old mice, consistent with the high brain expression of miRNAs and their active utilization in the modulation of protein expression in mouse brain (Noren Hooten *et al*., 2010; Inukai *et al*., 2012; van Spronsen *et al*., 2013). Among these 269 miRNAs, 55 were differentially expressed in the hippocampus between the 2‐ and 18‐month‐old mice. Among miRNAs regulated by age, miR‐34 is known to be upregulated in aged hippocampus and targets Sirt1 to regulate memory function (Zovoilis *et al*., 2011). In this study, we provided a second possible example of miRNA‐associated memory regulation through miR‐204. The functions of the remaining miRNAs differentially expressed in aged hippocampus remain to be determined.

We found that the total protein level of EphB2, a positive regulator of the axon guidance pathway, was 60% lower in the aged hippocampus than that in the young hippocampus, while the expression of the negative regulator of the axon guidance pathway, RhoA, was 150% higher in the aged hippocampus. These changes indicate that the axon guidance pathway was highly downregulated in the aged hippocampus. This too could contribute to age‐related memory deficits. Memory depends on the integrity of axonal pathways within the hippocampus and pathways connecting the hippocampus to regions such as frontal cortex, and these pathways also show age‐dependent deterioration (Rogalski *et al*., 2012). Age‐related loss of EphB2 may contribute to cognitive deficits by accelerating this deterioration.

miR‐204 was upregulated 2.5‐fold in the aged hippocampus (Fig. S5). We argue that one possible functional consequence of miR‐204 upregulation with age in the hippocampus is the downregulation of EphB2, with ensuing reduction in NR1 surface expression, suggesting that miR‐204 is involved in the regulation of surface expression level of NR1 through EphB2. The reduced total and surface expression of NR1 by miR‐204 in neurons could lead to a decrease in functional NMDA receptors at synapses, reducing synaptic plasticity and cognitive function.

It was recently reported that neuronal cells undergo senescence during the aging process (Jurk *et al*., 2012). In fully matured primary cultured hippocampal neurons (DIV24), we found that increased miR‐204 or decreased EphB2 was accompanied by increase in senescence markers such as SA‐β‐gal and p16. Loss of EphA3, one of the Eph family, was reported to be involved in inducing senescent phenotypes in nontransformed cells (Lahtela *et al*., 2013) and defects of EphA2, another member of Eph family, also induce senescence in cardiac progenitor cells (Goichberg *et al*., 2013). Our results indicate that reduced EphB2 expression in aged hippocampal neurons promotes senescence‐like phenotype. As miR‐204 targets EphB2, we argue that increased level of miR‐204 induces cellular senescence in the aged hippocampal neurons by repressing EphB2.

Why is then miR‐204 increased upon aging, which seems to have negative effect on an individual? One possible explanation is that the age‐associated increase in miR‐204 level is an evolutionary design to downregulate learning and memory capacity in aged animals (Mery *et al*., 2007). It is conceivable, for instance, that the threshold for synaptic plasticity is increased to stabilize existing synaptic circuits underlying older memory traces critical for survival (at the expense of new learning capacity). Alternatively, the increased level of miR‐204 in aged hippocampus may be a mechanism to coordinate hippocampal function with other regions of the aging brain.

Important future tasks will be testing whether exogenous miR‐204 in the hippocampus can induce early cognitive decline in mice and whether an antagomir of miR‐204 can restore EphB2 signaling and rescue NMDAR‐dependent LTP in aged mice. Indeed, several such antagomirs have been shown to rescue hippocampal pathology *in vivo* (Jimenez‐Mateos *et al*., 2015).

## Experimental procedures

### Animals and tissue collection

Male mice (C57BL/6J strain) at 2, 6 and 18 months of age were obtained from Korea Research Institute of Bioscience & Biotechnology‐Institutional Animal Care and Use Committee (KRIBB‐IACUC). All animals were maintained under ‘specific pathogen‐free’ conditions, and all studies were conducted according to the guidelines approved by the KRIBB‐IACUC. Mice without visible signs of tumorigenesis or disease were sacrificed, and their hippocampal tissues were immediately dissected and flash‐frozen in liquid nitrogen. Eight hippocampi from four mice were obtained from each age group.

### miRNA expression profiling

Small RNA sequencing was performed by a commercial service company (Macrogen Inc, Seoul, Korea) using Hi‐seq2000. The sequencing data from this study have been submitted to the NCBI Gene Expression Omnibus (GEO; http://www.ncbi.nlm.nih.gov/geo/) under accession number GSE74796.

### Pathway prediction of target genes

Possible targets for the differentially expressed miRNAs were predicted using TargetScan. The predicted target genes were used as input to the Database for Annotation, Visualization and Integrated Discovery, a KEGG functional analysis database (http://david.abcc.ncifcrf.gov/) with default parameter settings but restricted to mouse species.

### Biotinylation assay

Rat primary hippocampal neurons were transfected with the miR‐204 mimic, EphB2 siRNA or scramble control at 2 DIV and cultured until 7 DIV. The neurons were then placed on ice and rinsed twice with ice‐cold PBS. Cells were incubated with PBS containing 1 mg mL^−1^ sulfo‐NHS‐SS‐Biotin (Pierce Protein Research Products, Waltham, MA, USA) on ice for 30 min and then washed in PBS containing 100 mm glycine to remove unbound biotin. The cells were then washed again with PBS and incubated at 4 °C for 60 min with agitation in RIPA buffer containing protease inhibitors. The cells were lysed by brief sonication and centrifuged at 15 900 g for 15 min at 4 °C. The total protein concentration was measured in diluted aliquots of the cell lysate (1:9 in fresh RIPA buffer), and cell lysate samples (approximately 200 μg of protein/sample) were incubated with avidin agarose beads (Pierce Protein Research Products) at 25 °C for 60 min. The beads with bound biotinylated proteins were washed in PBS three times and boiled in 2× sample buffer and centrifuged to extract surface protein at 18 400 g for 5 min at 4 °C. Isolated protein (10 μg of surface and total lysate protein per gel lane) were electrophoretically separated and subjected to Western blot analysis as described in the Data S1. The levels of surface and total EphB2 and NR1 were quantified from band densities using nih imagej software (Bethesda, MD, USA).

### SA‐β‐gal assay and P16 immunostaining

Rat primary hippocampal neurons were transfected with the miR‐204 mimic, EphB2 siRNA or scramble control at 7 DIV and cultured until 24 DIV. Neurons were fixed with 4% paraformaldehyde and incubated overnight with SA‐β‐gal solution containing X‐gal. SA‐β‐gal‐positive cells were counted using light microscopy. For p16 immunostaining, 4% paraformaldehyde‐fixed hippocampal neurons were incubated overnight with p16 antibody Abcam (ab54210; Cambridge, UK) at 4 °C. After washing in PBS, the cells were incubated in Alexa 488‐conjugated secondary antibody (1:1000; Life Technologies Waltham, MA, USA) for 1 h at room temperature. p16‐positive cells were visualized using fluorescent microscopy.

## Funding info

This work was supported by the Institute for Basic Science (IBS‐R013‐D1 to HGN), DGIST R&D Program of the Ministry of Science, ICT & Future Planning (15‐BD‐06 to KK) and by National Research Foundation Basic Research Program grant 2012R1A1A2006838 (to KK) of the Ministry of Education, Science and Technology, Republic of Korea.

## Author contributions

C.P.D.M., K.K, H.G.N. and S.K.P. designed the research; C.P.D.M., K.K., B.P., J.Y.K., H.J.K., Y.J.K., H.C.K., H.H.L., J.H.P., J.H.J., H.S.L. and D.B. performed the research and analyzed the data; C.P.D.M., K.K. and H.G.N. wrote the paper.

## Conflicts of interest

The authors have declared that no competing interests exist.

## Supporting information


**Fig. S1** Cluster analysis of the differentially expressed miRNAs among the three age groups. The number in the parentheses indicates the total number of miRNAs up (U) or downregulated (D) in the cluster.

**Fig. S2** Differential expression of the miRNAs deduced from small RNA‐seq data was confirmed by quantitative PCR analysis. Darker and lighter histograms indicate qPCR and small RNA seq data respectively Shown are the average and standard deviation for each miRNA.

**Fig. S3** (A) Expression levels of ephrin ligand and receptor genes were examined by qRT–PCR from three independent hippocampal tissue samples. A single ephrin subtype (ephrinB3) and four Eph receptor subtypes (EphA1, EphA2, EphA4, and EphB2) exhibited significantly decreased mRNA expression levels in the oldest age group compared with those in the youngest (*P* < 0.05), ranging from 25% to 60%, whereas RhoA showed a 50% increase (*P* < 0.05). (B) The protein levels of EphB2, EphA4, RhoA, and tubulin (internal control) in the 2‐ and 18‐month‐old hippocampi as measured western blot analysis. EphB2 protein level was downregulated in the aged hippocampus compared with that in the young hippocampus. Two different animals for each of 2 months (2M) and 18 months (18M) were used for the analysis.

**Fig. S4** Effect of miR‐204 transfection on expression of wild‐type luciferase‐Ephb2 3′ UTR in HEK293 cells. SCR, scrambled miRNA control.

**Fig. S5** Expression level of miR‐204 was examined by qRT–PCR from independent hippocampal tissues (*n* = 6, 2 month; *n* = 3, 6 month; *n* = 6, 18 month). Shown are the average and standard deviation. *P < 0.05, M = month.

**Table S1** Total number of reads obtained from profiling the small RNA transcriptome in mouse hippocampus at 2, 6, and 18 months of age. (Non‐miRNA reads for 2M is 3 527 1412 756 663; 6M is 3 850 4103 111 722 and 18M is 5 156 4083 327 229).

**Table S2** Excel file for miRNA raw reads and normalized read counts.

**Table S3** Pathway annotations associated with the predicted targets of miRNAs upregulated in aged hippocampus.

**Table S4** List of 11 Eph/ephrin signaling components that are putative targets of hippocampal miRNAs.

**Data S1** Experimental procedures.
