# Supplementary figures and images for: miR‐204 downregulates EphB2 in aging mouse hippocampal neurons
Source: Aging Cell. 2016 Jan 22;15(2):380–8. doi: 10.1111/acel.12444 (PMC4783348; doi:10.1111/acel.12444)

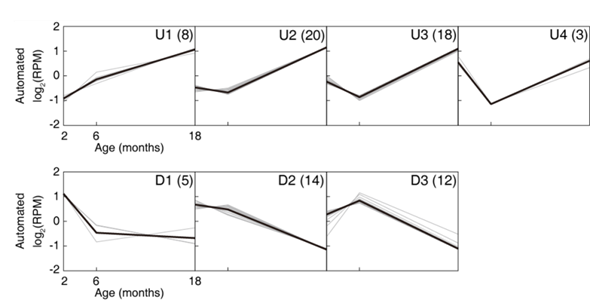

Supplement: Supplementary file 1 — Fig. S1 Cluster analysis of the differentially expressed miRNAs among the three age groups. The number in the parentheses indicates the total number of miRNAs up (U) or downregulated (D) in the cluster. [file ACEL-15-380-s001.tif]

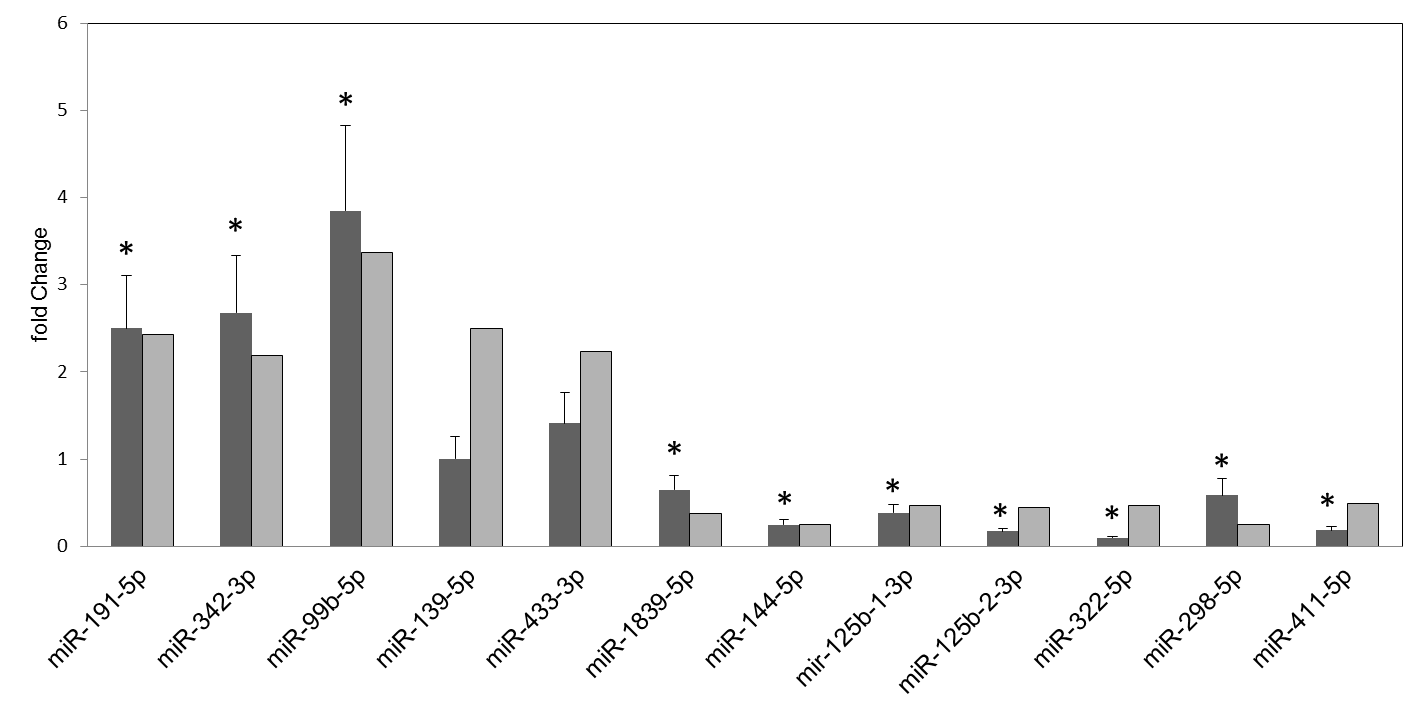

Supplement: Supplementary file 2 — Fig. S2 Differential expression of the miRNAs deduced from small RNA‐seq data was confirmed by quantitative PCR analysis. Darker and lighter histograms indicate qPCR and small RNA seq data respectively Shown are the average and standard deviation for each miRNA. [file ACEL-15-380-s002.tif]

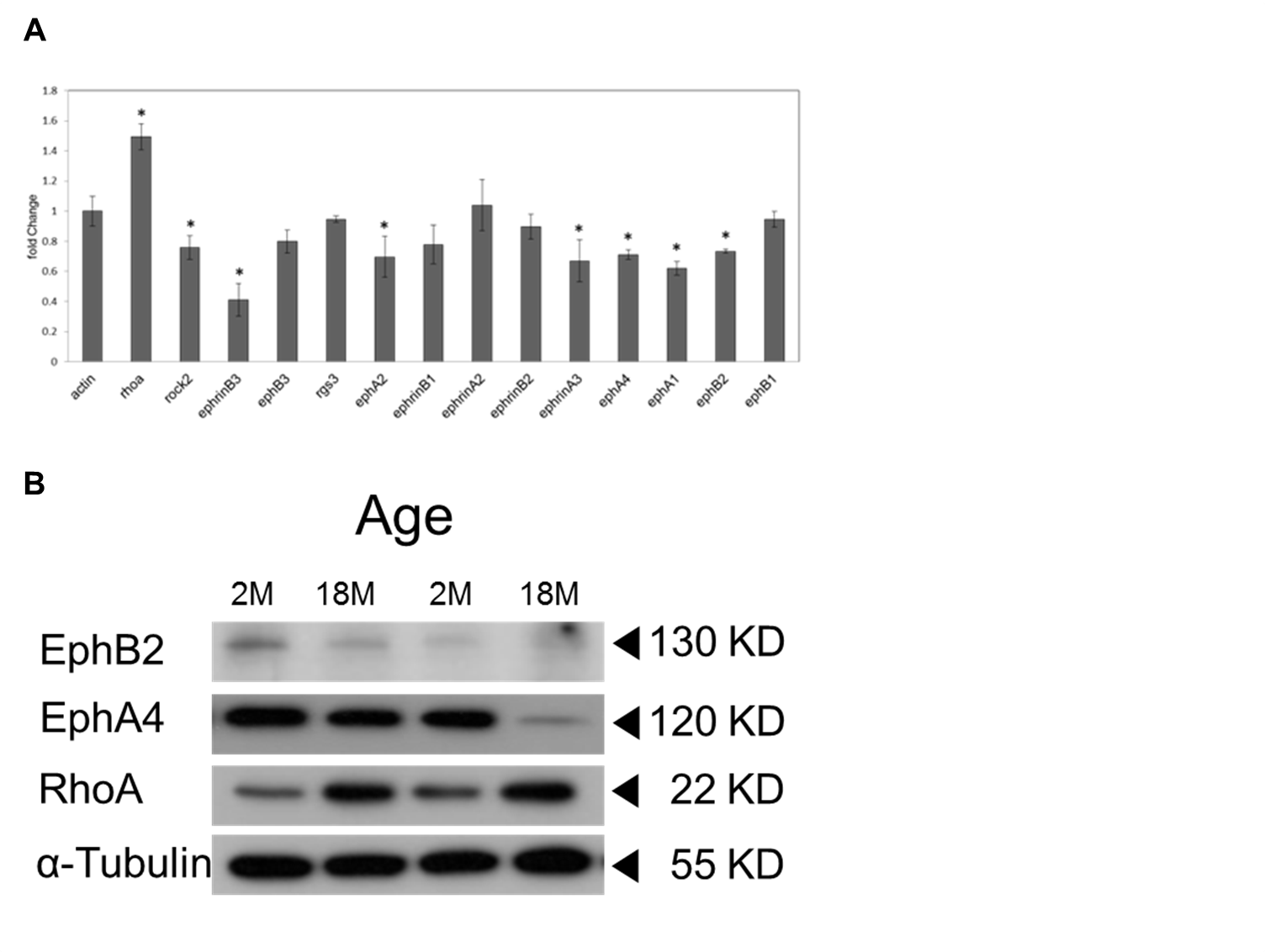

Supplement: Supplementary file 3 — Fig. S3 (A) Expression levels of ephrin ligand and receptor genes were examined by qRT–PCR from three independent hippocampal tissue samples. A single ephrin subtype (ephrinB3) and four Eph receptor subtypes (EphA1, EphA2, EphA4, and EphB2) exhibited significantly decreased mRNA expression levels in the oldest age group compared with those in the youngest (P < 0.05), ranging from 25% to 60%, whereas RhoA showed a 50% increase (P < 0.05). (B) The protein levels of EphB2, EphA4, RhoA, and tubulin (internal control) in the 2‐ and 18‐month‐old hippocampi as measured western blot analysis. EphB2 protein level was downregulated in the aged hippocampus compared with that in the young hippocampus. Two different animals for each of 2 months (2M) and 18 months (18M) were used for the analysis. [file ACEL-15-380-s003.tif]

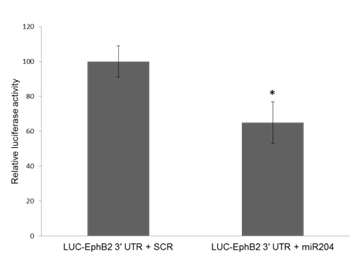

Supplement: Supplementary file 4 — Fig. S4 Effect of miR‐204 transfection on expression of wild‐type luciferase‐Ephb2 3′ UTR in HEK293 cells. SCR, scrambled miRNA control. [file ACEL-15-380-s004.tif]

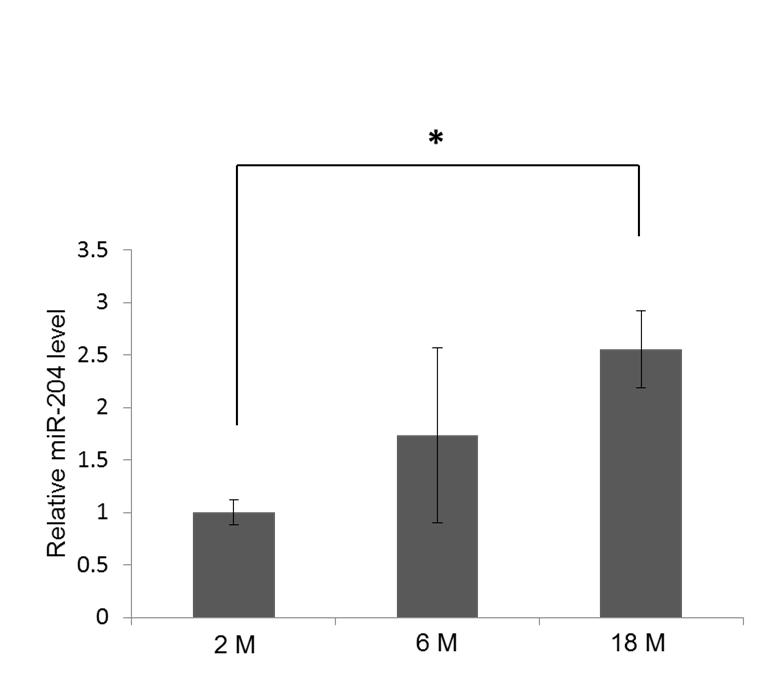

Supplement: Supplementary file 5 — Fig. S5 Expression level of miR‐204 was examined by qRT–PCR from independent hippocampal tissues (n = 6, 2 month; n = 3, 6 month; n = 6, 18 month). Shown are the average and standard deviation. *P < 0.05, M = month. [file ACEL-15-380-s005.tif]

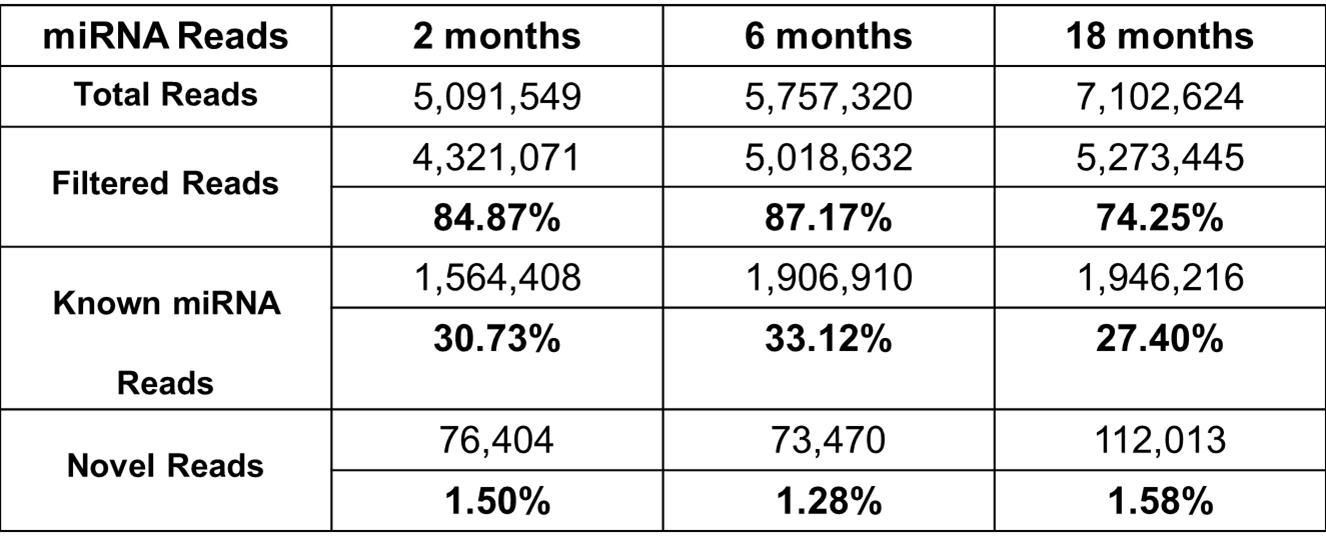

Supplement: Supplementary file 6 — Table S1 Total number of reads obtained from profiling the small RNA transcriptome in mouse hippocampus at 2, 6, and 18 months of age. (Non‐miRNA reads for 2M is 3 527 1412 756 663; 6M is 3 850 4103 111 722 and 18M is 5 156 4083 327 229). [file ACEL-15-380-s006.tif]

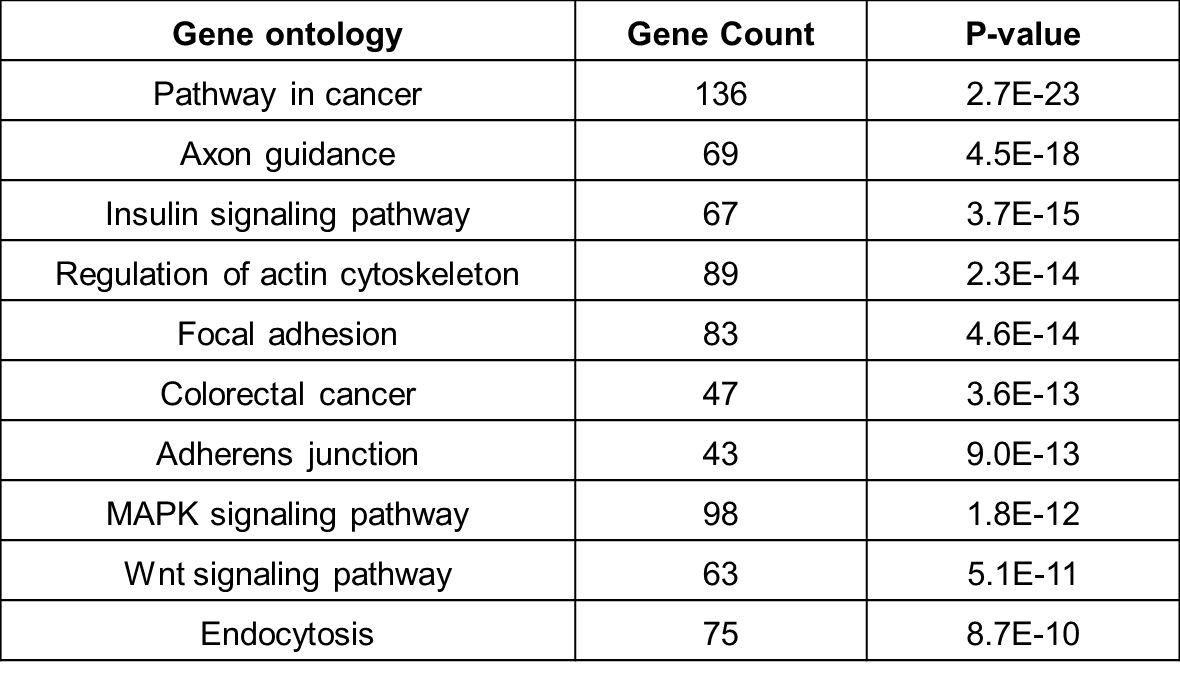

Supplement: Supplementary file 8 — Table S3 Pathway annotations associated with the predicted targets of miRNAs upregulated in aged hippocampus. [file ACEL-15-380-s008.tif]

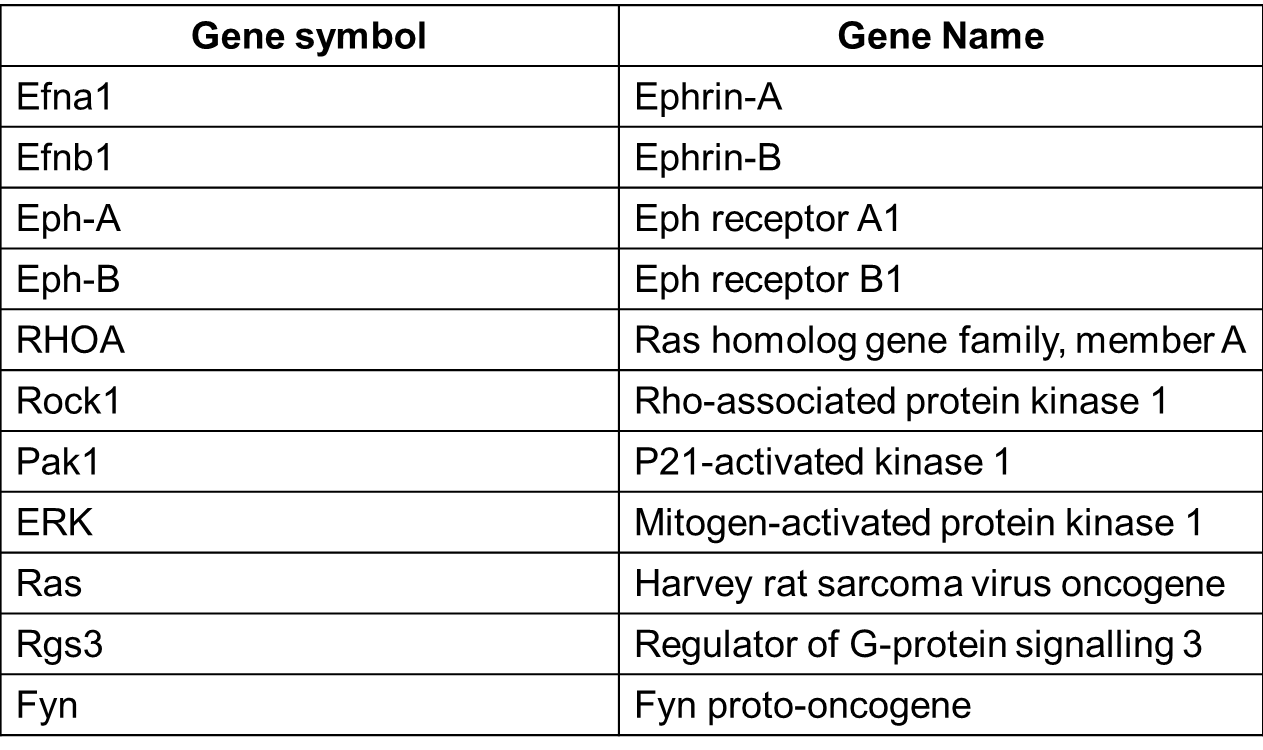

Supplement: Supplementary file 9 — Table S4 List of 11 Eph/ephrin signaling components that are putative targets of hippocampal miRNAs. [file ACEL-15-380-s009.tif]
