# Supplementary material for: miR‐204 downregulates EphB2 in aging mouse hippocampal neurons
Source: Aging Cell. 2016 Jan 22;15(2):380–8. doi: 10.1111/acel.12444 (PMC4783348; doi:10.1111/acel.12444)
Supplement: Supplementary file 10 — Data S1 Experimental procedures. [file ACEL-15-380-s010.docx]

**EXPERIMENTAL PROCEDURES**

**Micro RNA Extraction and cDNA library construction**

Small RNA was extracted using the Pure Link Micro-to-Midi total RNA Purification System kit **(Invitrogen,** Waltham, MA, USA**) and miRNeasy mini kit (Qiagen,** Venlo, Netherlands**).** Ten microgram of small RNA per gel lane was subjected to electrophoresis through a 15% TBE-urea PAGE gel. The fractions of the lanes containing RNA molecules between 18 and 30 nucleotides in length were excised. The small RNAs isolated from the gel slices were then attached with 5′ and 3′ adapters and were reverse transcribed using the Illumina Truseq kit, (Illumina, San Diego, California, USA). The resulting cDNAs were amplified by PCR using Illumina primers and were then subjected to polyacrylamide gel electrophoresis. The fractions of the lanes containing cDNA molecules between 60 and 80 nucleotides in length were excised for RNA extraction.

**Small RNA sequencing**

The small RNA sample libraries were subjected to single-end sequencing with a read length of 50 nucleotides. The resulting raw sequences were mapped against the reference mouse genome (Mus_musculus.NCBIM37.55) using the software programs Cufflinks and TopHat (John Hopkins University). The number of sequence reads that corresponded to known miRNAs was determined by perfect sequence matching to the database of known miRNAs (miR Base release version16, <http://www.mirbase.org/>). Raw data (the reads for each miRNA) were normalized to the total reads from each individual sample. We excluded miRNAs with fewer than 10 read counts per sample.

**Cell culture and transfection**

Dissociated hippocampal neurons were prepared from wild-type rat pups (P0). Neurons were plated on polylysine-coated glass coverslips (18 mm; Bellco Glass) in 12-well plates and were maintained in Neurobasal medium supplemented with B27 (Invitrogen), glutamine (Sigma-Aldrich, St. Louis, Missouri, USA), and penicillin–streptomycin (Sigma-Aldrich). Cells were seeded at 70,000 per well and were maintained in a humidified incubator with 5% CO_2_ at 37°C. Neurons were transfected at 2 DIV except for the luciferase assay, in which the neurons were transfected at 7 DIV by calcium phosphate precipitation.

**Western blot analysis**

Western blot analysis was performed as described in the Abcam® Western blotting protocol. Briefly, sample tissues were lysed in RIPA buffer, and aliquots (20−30 µg) of the whole lysate were electrophoresed on 5% sodium dodecyl sulfate/5%−20% gradient polyacrylamide gels (SDS-PAGE), and electrotransferred to nitrocellulose membranes. The membranes were blocked in Tris-buffered saline containing 5% nonfat milk and were then hybridized with the primary antibody followed by the secondary antibody prepared in the same blocking solution. Immunolabelling was visualized using the enhanced chemiluminescence detection system. The antibodies against α-tubulin, RhoA, NMDA receptor subunit NR1, EphB2, and EphA4 were obtained from AbClon (AbC-2001, **Seoul, Korea**), Abcam (ab68826, Cambridge, UK), Life Technologies (32-0500, Waltham, MA, USA), R&D systems (H00002048-M03, Minneapolis, MN, USA), and Abnova (H00002043-M02, Taipei, Taiwan), respectively. Protein band intensity was quantified using ImageJ software (ImageJ 1.46).

**Immunocytochemistry**

Cells were fixed in 4% paraformaldehyde/4% sucrose PBS for 10 min at room temperature, washed three times in PBS, permeabilized in PBS containing 0.1% triton X-100 for 10 min at room temperature, and blocked in PBS containing 5% BSA. Fixed cultures were incubated in rabbit anti-GFP (Abcam: 1:1000, Cambridge, UK) primary antibody at 4°C overnight. After three washes in PBS containing 1% BSA, the cells were incubated in Alexa 488-conjugated secondary antibody (Life Technologies 1:1000) for 1 h at room temperature.

**Quantitative real-time reverse transcriptase–polymerase chain reaction analysis**

Total RNA was isolated from mice hippocampi (both hemispheres) using TRIzol reagent (Invitrogen Life Technologies, Waltham, MA, USA) according to the manufacturer’s recommendations. All samples showed A260/A280 and A260/A230 ratios over 2 and 1.9, respectively. RNA integrity was further examined using the Bio-analyzer Nano 6000 chip (Agilent Technologies), and all samples had an RNA Integrity Number over 8. Total RNA and small RNA samples were converted to cDNAs with PrimeScript™ RT reagent Kit (Promega ImPromm-II Reverse Transcription system, Fitchburg, Wisconsin, USA) and NCode™ miRNA First strand cDNA module (Invitrogen) respectively. One microgram sample of total or small RNA was used to generate a cDNA template. Quantitative real-time PCR was performed using the Bio-Rad real-time PCR system (CFX-96). Primer design was performed using Primer Blast (<http://www.ncbi.nlm.nih.gov/tools/primer-blast/> or <http://pga.mgh.harvard.edu/primerbank/>). The total reaction volume was 20 μL. All reactions were performed on a 96-well plate with SYBR® Green Mix. Target mRNA levels were normalized against actin mRNA and sno202 small RNA (control RNAs) for mRNA and miRNA quantification, respectively, according to the following formula: [2 ^−(CT target − CT control)^] × 100%, where CT is the threshold cycle.

**Statistical analyses**

All statistical data analyses were conducted using Microsoft® Excel 2010.
